# Pooled Prevalence and Determinants of Completion of Maternity Continuum of Care in Sub-Saharan Africa: A Multi-Country Analysis of Recent Demographic and Health Surveys

**DOI:** 10.3389/fgwh.2022.869552

**Published:** 2022-05-25

**Authors:** Melaku Hunie Asratie, Daniel Gashaneh Belay

**Affiliations:** ^1^Department of Women's and Family Health, School of Midwifery, College of Medicine and Health Sciences, University of Gondar, Gondar, Ethiopia; ^2^Department of Human Anatomy, College of Medicine and Health Sciences, University of Gondar, Gondar, Ethiopia; ^3^Department of Epidemiology and Biostatistics, Institute of Public Health, College of Medicine and Health Sciences, University of Gondar, Gondar, Ethiopia

**Keywords:** Africa, completion, DHS, maternity continuum of care, multilevel

## Abstract

**Background:**

Improving the coverage of completion of the maternity continuum of care is the priority area of sub-Saharan African countries to achieve the sustainable development goal. Despite this, information is scant about the pooled prevalence of completion of the maternity continuum of care and its determinants in sub-Saharan African countries. Therefore, this study aimed to assess the pooled prevalence of completion of the maternity continuum of care and its determinants among women in sub-Saharan African countries.

**Methods:**

The study was conducted based on Demographic and Health Survey (DHS) data from 33 sub-Saharan African countries from 2010 to 2020. The total sample size of 337,297 postpartum period women with children aged 0–36 months (about 3 years) was employed in the analysis by STATA version 14. A multilevel logistic regression model was fitted, and intraclass correlation coefficient (ICC), median odds ratio (M*OR*), proportion change in variance (PCV), and deviance were used for model fitness and comparison. Adjusted odds ratio (A*OR*) with its 95% confidence interval (*CI*) was presented. Variables with a value of *p* < 0.05 were declared significant determinants of completion of the maternity continuum of care.

**Results:**

The pooled prevalence of completion of the maternity continuum of care was 35.81%; [95% CI, 35.64%, 35.9%]. Higher educational level [A*OR* = 3.62; 95% *CI* 2.25, 4.46], wanted pregnancy [A*OR* = 2.51; 95% *CI* 1.82, 3.12], history of terminated pregnancy [A*OR* = 3.21; 95% *CI* 2.86, 4.21], distance to the nearby health facility [A*OR* = 2.11; 95% *CI* 1.68, 2.36], women as primary decision maker [A*OR* = 2.15; 95% *CI* 2.02, 2.87], nouse of traditional medication during pregnancy [A*OR* = 1.01; 95% *CI* 1.00, 1.45], antenatal care (ANC) visit within second trimester [A*OR* = 2.76; 95% *CI* 2.01, 3.47], informed about pregnancy complication [A*OR* = 2.73; 95% *CI* 2.10, 3.21], healthcare provider support [A*OR* = 1.77; 95% *CI* 1.02, 2.44], and being eastern and western African [A*OR* = 1.55; 95% *CI* 1.11, 2.44 and A*OR* = 2.01; 95% *CI* 1.88, 2.76, respectively] were determinant factors of completion of maternity continuum of care.

**Conclusions:**

The completion of the maternity continuum of care in sub-Sahara African countries was low. Emphasis on women's education, type, history of pregnancy, distance to the nearby health facility, region, assessing the exposure of traditional medication, and informed about pregnancy complications, healthcare provider's support can improve the prevalence of completion of the maternity continuum of care.

## Background

In developing regions, especially in sub-Saharan Africa, pregnancy-related preventable morbidities take the lion's share of the causes of maternal, neonatal, and child death (1, 2). Consolidation of qualified maternity care during pregnancy, childbirth, and the postpartum period is the priority agenda of the region and the world at large to achieve the sustainable development goal (3). There are global targets for survival for maternal mortality (70 per 100,000 live births and country not above 140), neonatal mortality (12 per 1,000 live births), and stillbirths (12 per 1,000 births) by 2030 (4). In line with the target, there has been substantial improvement in maternal healthcare services in sub-Sahara African countries since 2015 (4, 5). Though there have been some improvements, sub-Saharan Africa is known as the highest maternal mortality ratio (542 per 100,000 live births) region in the world (1). However, we are still a long way from achieving our goal of eliminating preventable maternal death, stillbirth, and neonatal death by 2030 (6). Recently published data show that there is a need to double the average annual rate of decline in sub-Saharan Africa to reach the 2030 target (1, 7).

Currently, there is a new roadmap entitled cultivating the culture of completion of the maternity continuum of care (each pregnant woman has four or more antenatal care (ANC) contacts, birth attended by skilled health personnel, and receives early routine postnatal care within 2 days), as this will determine the course of health and life of women, neonates, and child to achieve the sustainable development goal by 2030 (6). Improvement in the prevalence of completion of the maternity continuum of care is especially important to reduce the unresponsive neonatal mortality rate with the existing programs (8). Effective health measures at birth and during the first 7 days of the postpartum period can prevent two-thirds of newborn deaths (9). For instance, ensuring a clean birth, umbilical cord care, and immediate breastfeeding practice can avoid neonatal death due to infection (10). Therefore, improving the coverage of completion of the maternity continuum of care is one of the possible strategies to achieve an ambition of ending preventable causes of maternal and neonatal death (11–13). Shreds of evidence show that completion of the maternity continuum of care can be insured by the positive health-seeking behaviors of pregnant women, strong bond with the culture of the society, and healthcare providers (14). For this, adequate information is particularly important in sub-Saharan African countries.

Though varieties of individual studies are present (8, 15, 16), there is no single study that incorporates all countries in the sub-Sahara African region regarding the pooled prevalence and predictor variables (maternal age, residence, women's educational level, relation to household head, sex of household head, media exposure, wealth index, marital status, husband/partner's educational status, parity, number of children under 5 years of age, wanted pregnancy, history of terminated pregnancy termination, distance to the nearby health facility, health insurance coverage, sex preference, desire for future birth, decision-maker on contraceptive use, decision-maker for the maternity continuum of care, birth order, history of traditional medication, time of ANC visit, the content of ANC, informed about pregnancy complication, site of ANC visit, referral condition, and healthcare provider support).

Therefore, this study aimed to assess the pooled prevalence and determine the predictors of completion of maternity continuum of care in sub-Sahara African countries. Hence, this study attempted to fill this gap, which may help planners and policymakers design effective strategies to directly act on significant factors to increase the completion of the maternity continuum of care in the region. Furthermore, the findings of this study might be important to advance and expand agendas to go beyond survival with a view to maximizing the quality of maternal healthcare services and the potential of maternal, neonatal, and child health outcomes in sub-Saharan Africa.

## Methods

### Study Design, Area, and Period

A cross-sectional study design was employed based on the Demographic and Health Survey (DHS) 2010–2020 data from 33 sub-Saharan African countries. Sub-Saharan Africa is a region with high maternal and under-5 month infant mortality. In the region, maternal healthcare services are the priority area to achieve sustainable development goals. The DHS is the nationally representative survey that collects data on basic health indicators, such as maternal, neonatal, and child healthcare services, fertility, morbidity, and mortality. Each country's survey consists of different datasets, such as women, men, children, household, and birth datasets.

### Data Source and Sampling Procedure

The data were obtained by registering for the measure DHS program by authorized users after receiving online request from http://www.dhsprogram.com website. The study participants were selected by a two-stage stratified sampling technique. In the first stage, enumeration areas (EAs) were randomly selected, while in the second stage, households were randomly selected. For this study, the Children's Record (KR) dataset was used, and the data were first weighted (mathematically adjusted) by using sampling weight (v005), primary sampling unit (v021), and strata (v022) to keep the true distribution of postpartum period women with children aged 0–36 months (about 3 years) in the region. The total weighted sample size of this study was 337,297 postpartum period women with children aged 0–36 months (about 3 years). The detailed sampling procedure is presented elsewhere (17).

### Variables of the Study

The dependent variable of this study was “completion of maternity continuum of care,” categorized dichotomously as “yes/no.” A woman who completed the maternity continuum of care was categorized as “yes” and otherwise as “no.” Therefore, the Ith mother Yi's response variable was measured as a dichotomous variable with a possible value of Yi = 1 if the Ith mother completed the maternity continuum of care and Yi = 0 if the Ith mother does not complete the maternity continuum of care.

Independent variables were of two types: level one (individual-level variables) and level two (community-level variables). Level one variables include maternal age, residency, women's educational status, media exposure, wealth index, marital status, parity, number of children under 5 years of age, wanted pregnancy, history of terminated pregnancy, distance to the nearby health facility, health insurance coverage, sex preference, desire for children, decision-maker to attend maternal healthcare services, birth order, traditional medication use during pregnancy, gestational ANC start, the content of ANC services, informed about pregnancy complication, site for ANC services, healthcare provider support during ANC, and level 2 variables included residence and region (Table 1).

**Table 1:** The detailed variables code and recode of 33 sub-Sahara African countries Demographic and Health Survey (DHS).

**Age of women's**	**Re-coded in to four categories with a value of “1” for 15–19, “2” for 20–34, “3” for 35–49. In the data set this variable was continuous data**.
Residence	The variable place of residence was recorded as “rural” and “urban” in the dataset and used without change for this study.
Marital status	Never in union, currently in union/living with a man, and formerly in union/living without a man in the data set and we have used by recoding 1=single, 2= married.
Women's level of education	The variable women's education level was recorded as no education, primary, secondary, and higher in the dataset and we used by recoding no education=1, primary-secondary=2, and higher=3.
Wealth status	It was coded as “poorest”, “poorer”, “Middle”, “Richer”, and “Richest” in the DHS data set. For this study we recoded it in to three categories as “poor” (includes the poorest and the poorer categories), “middle”, and “rich” (includes the richer and the richest categories).
Parity	In the dataset this variable was continuous data. We re-coded in to three categories with a value of “1” for 1, “2” for 2, 3 and4, and “3” for ≥5.
ANC visit	In the data set no ANC, 1, 2, 3…20. We have recoded in to No = no ANC and Yes = else.
Place of delivery	In the data set other home, Government hospitals, Government health centers', Government health post, Other clinics, Private hospital/clinic, Other private medical clinic, and Others. We have used by recoding as “home” and “health facility”.
Post-natal care	In the data set Yes/No. We have recoded in to 1 = Yes and 2 = No.
Completion of maternity continuum of care	Yes/No. Generated from ANC visit Yes +health facility delivery +post-natal care Yes.

### Operational Definitions

#### Completion of the Maternity Continuum of Care

A woman who (i) had at least one antenatal care visit during pregnancy, (ii) delivered at a health facility, and (iii) had postnatal care within 6 weeks (about 1 and a half months) of the postpartum period was considered to have completed the maternity continuum of care and was coded as “Yes.” Otherwise, losing one of the three levels was considered an not completing the maternity continuum of care and was coded as “No” (8).

#### Components of Routine Antenatal Care Services

This was measured based on eight essential elements of antenatal care services: blood pressure measurement, blood sample collection, urine sample collection, weight measurement, tetanus toxoid (TT2+) vaccination, iron folate (90+) supplementation, health education on danger signs and nutrition, and HIV testing. Information on these eight items of ANC contents was derived from the response of the question “As part of your ANC during this pregnancy, was any of the following done at least once? Was your weight measured? Was your blood pressure measured? …….”. The answers were recorded as “Yes or No.” It is possible that a single mother may have a urine test, blood test, measurement of weight, or blood pressure several times during the same pregnancy. However, as the mother was asked to report any action at least once, the response to any action was recorded as a single action. Based on responses, we have created a composite index of the ANC content as our second outcome variable which comprises a simple count of the number of elements of care received. Finally, the outcome variable is dichotomized into incomplete if a woman gets less than eight services = 0 and complete if a woman gets all eight elements = 1 (18, 19).

#### Postnatal Care Visit

Health facility visit to check the health status of the mother and newborn (20).

### Data Management and Analysis

We pooled the data from 33 sub-Saharan African countries. To ensure the survey's representativeness, the data were weighted by sampling weight, strata, and primary sampling unit prior to analysis. Tables and charts were used to report both individual and community-level factors and the pooled prevalence of completion of the maternity continuum of care. Multilevel multivariable logistic regression was fitted by considering the cluster variability.

#### Model Building

In this study, we fit four models: null model without predictor variables, model one with individual-level factors, model two with community-level factors, and model three that includes both individual and community-level factors. The STATA command **melogit** was used to fit these models. Finally, according to the output, the best fit model of the current study was model three with [individual + community-level factors] (**Table 5**).

#### Parameter Estimation Methods

In the current study, the fixed-effect model was used to estimate the statistical association between the exploratory variables [individual and community level] and the completion of the maternity continuum of care. Bivariable analysis was done and variables with a *p* ≤ 0.2 were considered as a candidate for multilevel multivariable analysis. In a multivariable analysis, adjusted odds ratios (A*OR*s) with respect to a 95% confidence interval (*CI*) were reported, and variables with a value of *p* < 0.05 were considered statistical significant with the completion of the maternity continuum of care.

The random-effects model measures the variation of completion of the maternity continuum of care across clusters expressed by intraclass correlation coefficient [ICC], which quantifies the degree of variation in completion of the maternity continuum of care between clusters. In the current study, 18.2% of the variation was due to clustering effect; Median odds ratio [M*OR*], that is, the median value of *OR* between low and high *OR* completion of the maternity continuum of care when we randomly picked out two clusters, was 2.33 [2.28, 2.52] for the current study, and the percentage change in variance [PCV] indicates the proportion of the total observed individual variation of completion of the maternity continuum of care that is attributable to between-cluster variations, and the final model of the current study was with a PCV of 57%.

Deviance [-2log-likelyhood ratio] was used for model comparisons and the final model with the smaller deviance of 125,404 was the best fit model for the current study (**Table 5**).

## Results

### Socio-Demographic Characteristics of the Study Participants in Sub-Saharan Africa

Among the 337,297 participants, 238,518 [70.71%] of the women were between the age group of 25 and 34 years, 315,265 [93.47%] of them were married, and 201,244 [59.66%] had primary-secondary education. Off all participants, 231,776 [68.72%], 159,112 [47.17%], 214,559 [63.61%], and 129,410 [38.37%] were rural in residency, poor wealth status, have media exposure, and from West Africa region, respectively (Table 2).

**Table 2:** Socio-demographic characteristics of the participants in sub-Sahara Africa.

**Variable**		**Weighted**	**Percentage**
		**frequency**	
		**(*n* = 337297)**	**(%)**
Age of women's	15–24	20,406	6.05
	25–34	238,518	70.71
	35–49	78,373	23.24
Marital status	Single	22,032	6.53
	Married	315,265	93.47
Women's level of education	No education	125,109	37.09
	Primary –secondary	201,244	59.66
	College and above	10,944	3.20
Residency	Rural	231,776	68.72
	Urban	105,521	31.28
Wealth status	Poor	159,112	47.17
	Middle	66,051	19.58
	Rich	112,134	33.25
Media exposure	No	122,738	36.39
	Yes	214,559	63.61
Region	East Africa	116,725	34.61
	Central Africa	48,412	14.35
	West Africa	129,410	38.37
	South Africa	42,750	12.67

### Maternal Healthcare Services Related Factors of Women in Sub-Saharan African Countries

From all the participants of the current study, women with distance to the nearby health facility, not a big problem was 210,950 [62.54%], not covered by health insurance was 284,099 [84.23%], and 41,080[12.18%] women were the primary decision-maker to attend the maternity continuum of care. Among all participants 211,502 [62.70%], 252,828 [74.96%], 267,017 [79.16%], 169,797 [50.34%], and 285,758 [84.72%] of participants had not used traditional medication during pregnancy, ANC visits started above second trimester, they got complete components of routine ANC, a site for ANC services from the government health facility, and they had healthcare provider support during ANC services (Table 3).

**Table 3:** Maternal health care services related factors of women in sub-Saharan African.

**Variables**	**Categories**	**Weighted**	**Percentage**
		**frequency**	
		**[*N* = 337297]**	**(%)**
Distance to the nearby	No a big problem	210,950	62.54
health facility	A big problem	126,347	37.46
Covered by health	No	284,099	84.23
insurance	Yes	53,198	15.77
Decision maker to	Others	191,118	56.66
attend maternity	Jointly with husband	105,099	31.16
continuum of care	Independently by women	41,080	12.18
Traditional medication	No	211,502	62.70
use during pregnancy	Yes	125,795	37.30
Gestational age for	Within second trimester	84,469	25.04
ANC start first	Above second trimester	252,828	74.96
Content of antenatal	Not complete	70,280	20.84
care	Complete	267,017	79.16
Site for ANC	Government health facility	169,797	50.34
	Non-government	167,500	49.66
Health care provider	Not	51539	15.28
support during ANC	Yes	285,758	84.72

### Obstetrical Related Factors of Women in Sub-Saharan African Countries From DHS 2010–2020

Among 337,297 participants, 239,337 [70.96%], 274,076 [81.26%], and 306,423 [90.85%] had 1–4 children, no history of a terminated pregnancy, and wanted pregnancy, respectively. Off all participants, 334,787 [99.26%], 214,758 [63.67%], 262,790 [77.91%], and 301,491 [89.37%] of them were grand multipara, with male sex preference, birth order other than one, and being informed about pregnancy complications during pregnancy, respectively (Table 4).

**Table 4:** Obstetrical related factors of women in sub-Saharan African countries DHS 2010–2020.

**Variables**	**Categories**	**Weighted**	**Percentage**
		**frequency**	**(%)**
Number of under 5	No	2,503	0.74
children	1–4	239,337	70.96
	5& above	95,457	28.30
History of terminated	No	274,076	81.26
pregnancy	Yes	63,221	18.74
Wanted pregnancy	Yes	306,423	90.85
	No	30,874	9.15
Parity	Primiparous	621	0.18
	Multiparous	1,889	0.56
	Grand multipara	334,787	99.26
Sex preference	Male	214,758	63.67
	Female	122,539	36.33
Desire for more children	Yes	214,758	63.67
	Undecided	20,025	5.94
	No more children	102,514	30.39
Birthorder1	Yes	74,507	22.09
	No	262,790	77.91
Told about pregnancy	Yes	301,491	89.38
complication	No	35,806	10.62

### Pooled Prevalence of Completion of the Maternity Continuum of Care in Sub-Sahara Africa 2010–2020 DHS Data

**Figure 1:**
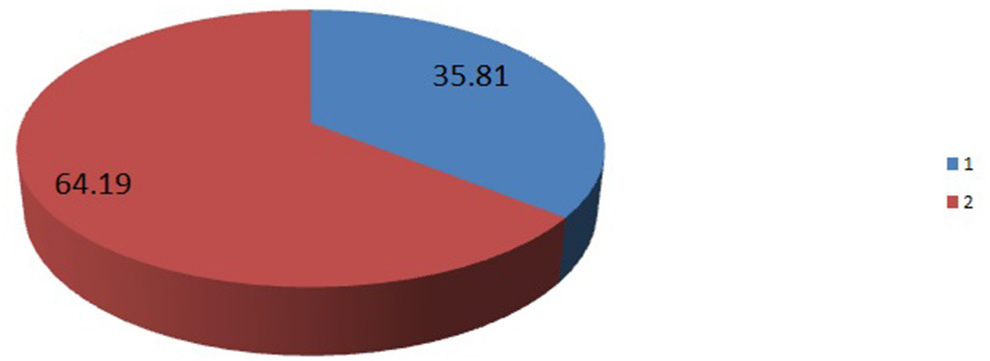
The pooled prevalence of completion of maternity the continuum of care in 33 sub-Sahara African countries from 2010 to 2020 Demographic and Health Survey (DHS) report. 1, the pooled prevalence of completion of maternity continuum of care. 2, prevalence of not complete maternity continuum of care.

**Figure 2:**
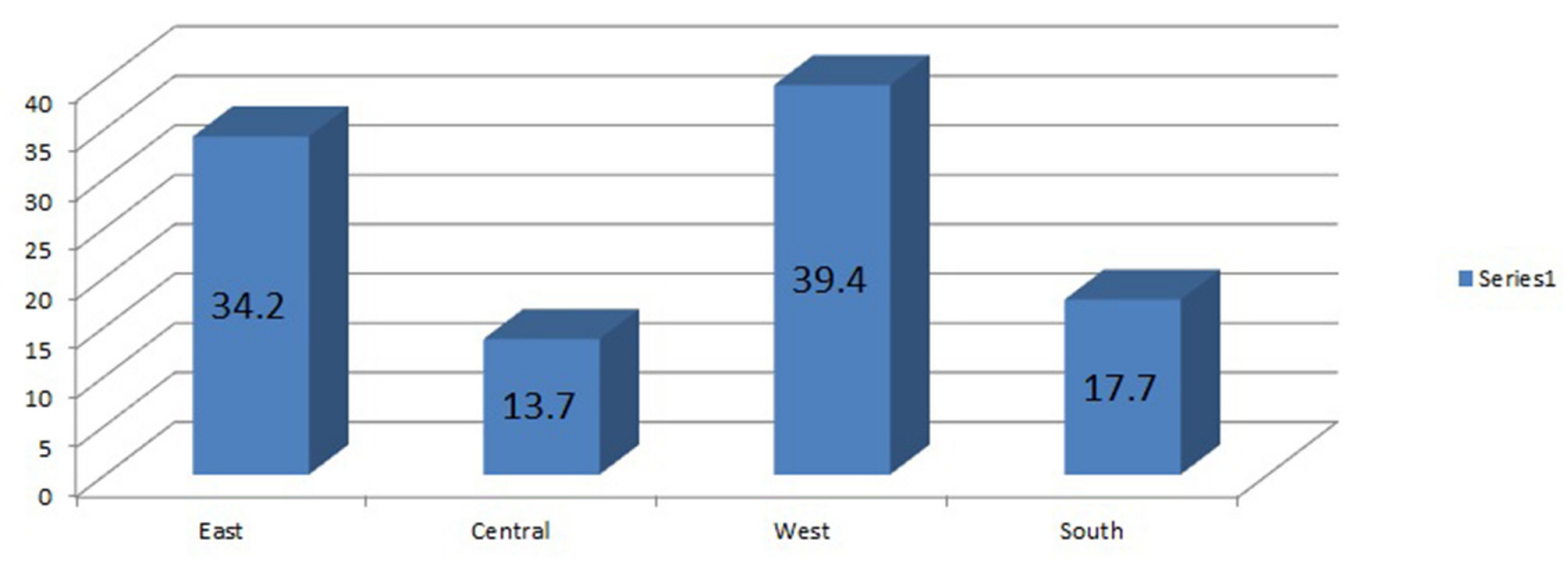
The prevalence of completion of the maternity continuum of care among 4 sub-Sahara African regions from 2010 to 2020 DHS report.

The analysis was done based on 33 sub-Saharan African countries (Burundi, Comoros, Ethiopia, Kenya, Malawi, Mozambique, Rwanda, Tanzania, Uganda, Zambia, Zimbabwe, Angola, Cameroon, Chad, DR Congo, Congo, Gabon, Benin, Burkina Faso, Ivory Coast, Gambia, Ghana, Guinea, Liberia, Mali, Niger, Nigeria, Senegal, Sierra Leone, Togo, Lesotho, Namibia, South Africa). All those 33 countries are grouped into four regions, i.e., East African countries, Central African countries, West African countries, and Southern African countries (Table 5). The pooled prevalence of completion of the maternity continuum of care in sub-Sahara African countries from 2010 to 2020 was found to be 35.81%; 95% [35.64%, 35.9%] (Figure 1). Surprisingly, the prevalence of completion of the maternity continuum of care varied among different regions of sub-Sahara Africa, with a higher report from West Africa region 39.4% and the lowest report was from the Central Africa region 13.7% (Figure 2).

**Table 5:** Sub-Saharan Africa countries with recent DHS report from 2010/11 to 2019/20.

		**Standard**
**Regions**	**Countries**	**DHS year**
East Africa countries	Burundi	2016/17
	Comoros	2012
	Ethiopia	2016
	Kenya	2014
	Malawi	2015/16
	Mozambique	2015
	Rwanda	2019/2020
	Tanzania	2015/16
	Uganda	2016
	Zambia	2018
	Zimbabwe	2015
	Angola	2015/16
	Cameroon	2018
Central Africa countries	Chad	2014/15
	DR Congo	2013/14
	Congo	2011/12
	Gabon	2012
	Benin	2017/18
	Burkina Faso	2011
	Ivory Coast	2011/12
	Gambia	2019/20
	Ghana	2014
West Africa countries	Guinea	2018
	Liberia	2019/20
	Mali	2018
	Niger	2012
	Nigeria	2018
	Senegal	2019
	Sierra Leone	2019
	Togo	2013/14
Southern Africa countries	Lesotho	2014
	Namibia	2013
	South Africa	2016

### Predictors of Completion of the Maternity Continuum of Care in Sub-Sahara African Countries

A multilevel logistic regression model was fitted, and a total of 20 variables were candidates for multivariable multilevel analysis. In the multivariable multilevel analysis, one community-level factor (region), and 9 individual-level factors (women's educational status, wanted pregnancy, history of a terminated pregnancy, distance to the nearby health facility, decision-maker to attend maternal healthcare services, use of traditional medication during pregnancy, gestational age at ANC start, told pregnancy complication, and healthcare provider support during ANC) were statistically significant with the completion of the maternity continuum of care.

Women with higher educational status are 3.61 times more likely to complete the maternity continuum of care (A*OR* = 3.61; 95% *CI* 2.25, 4.46) compared with those who have no formal education. The odds of completion of the maternity continuum of care were 2.51 times higher among women with a wanted pregnancy (A*OR* = 2.51; 95% *CI* 1.82, 3.12) as compared to women with pregnancy was not wanted. Women with a history of terminated pregnancy are 3.21 times more likely to complete the maternity continuum of care (A*OR* = 3.21; 95% *CI* 2.86, 4.21) compared with those who have no history of terminated pregnancy. The odds of completion of the maternity continuum of care 2.11 times higher among women whose distance to the nearby health facility was not a big problem (A*OR* = 2.11; 95% *CI* 1.68, 2.36) compared with women whose distance to the nearby health facility was a big problem. Women who can decide independently to attend maternal healthcare services are 2.15 times more likely to complete the maternity continuum of care (A*OR* = 2.15; 95% *CI* 2.02, 2.87) compared with those whose decisions were made by others. The odds of completion of the maternity continuum of care are 1.01 times higher among women who have not used traditional medication during pregnancy (A*OR* = 1.01; 95% *CI* 1.00, 1.45) compared to women who have used traditional medication during pregnancy.

Women who start ANC visits within the second trimester are 2.76 times more likely to complete the maternity continuum of care (A*OR* = 2.76; 95% *CI* 2.01, 3.47) as compared with women who start ANC above the second trimester. The odds of completion of the maternity continuum of care were 2.73 times higher among women told about pregnancy complications (A*OR* = 2.73; 95% *CI* 2.10, 3.21) compared with women who had not been told about pregnancy complications. Women with healthcare provider support during the ANC services are 1.77 times more likely to complete the maternity continuum of (A*OR* = 1.77; 95% *CI* 1.02, 2.44) compared with women who have no healthcare provider support during ANC. Being eastern, and western region of sub-Sahara Africa 1.55 and 2.01 times higher completion of maternity continuum of care (AOR=1.55; 95% CI 1.11, 2.44), and (AOR=2.01; 95% CI 1.88,2.76) as compared to women with a region of South Africa respectively (Table 6).

**Table 6:** Multilevel logistic regression analysis for the assessment of predictors of completion of the maternity continuum of care in sub-Sahara Africa, DHS 2010–2020 data.

**Variables**		**Null model**	**Model I**	**Model II**	**Model III**
			**AOR(95% CI)**	**AOR(95% CI)**	**AOR(95% CI)**
Age of women's	15 to 24		1		1
	25 to 28		0.79 (0.66, 0.95)		1.80 (0.67, 1.96)
	29 to 32		0.77 (0.62, 0.96)		2.79 (0.63, 2.98)
	33 to 49		0.66 (0.52, 0.83)		1.67 (0.83, 1.85)
Number of under 5 age	5		1		1
Children alive	<5		1.51 (1.38, 1.69)		1.02 (0.88, 1.69)
Marital Status	No married		1		1
	Married		1.33 (1.03, 1.48)		1.84 (0.93, 2.49)
Media exposure	No		1		1
	Yes		1.15 (0.99, 1.34)		1.15 (0.99, 1.34)
Women's level of	No education		1		1
educational	Primary		1.13 (1.04, 1.52)		1.01 (0.89, 1.38)
	Secondary		1.64 (1.34, 2.36)		1.87 (0.78, 2.26)
	Higher		2.50 (1.79, 3.47)		3.61 (2.25, 4.46)^***^
Parity	1 to 2		1		1
	3 to 4		1.44 (1.21, 1.72)		1.21 (0.91, 1.73)
	5		1.63 (1.31, 2.03)		1.41 (0.89, 2.00)
Pregnancy was wanted	No		1		**1**
	Yes		1.34 (1.12, 1.86)		2.51 (1.82, 3.12)^**^
History of terminated	No		1		1
pregnancy	Yes		2.12 (1.68, 2.86)		3.21 (2.86, 4.21)^***^
Wealth status	Poor		1		1
	Middle		0.91 (0.77, 1.07)		0.94 (0.80, 1.11)
	Rich		1.07 (0.91, 1.25)		1.15 (0.97, 1.37)
Distance to the nearby	Not a big problem		1		1
health facility	A big problem		2.42 (1.98, 3.26)		2.11 (1.68, 2.36)^***^
Content of antenatal care	Not full content		1		1
	Full content		1.23 (1.02, 2.31)		1.03 (0.78, 1.54)
Decision maker to attend	Other than women		1		1
maternal health	Jointly		1.45 (1.24, 2.01)		1.02 (0.86, 1.65)
	Independently by women		2.34 (1.86, 2.89)		2.15 (2.02, 2.87)^***^
Health insurance	No		1		1
	Yes		0.75 (0.54, 1.05)		0.82 (0.58, 1.16)
Use of medication during	Yes		1		1
pregnancy	No		1.24 (1.01, 2.56)		1.01 (1.00, 1.45)^***^
Antenatal care start	Above second trimester		1		1
	Within second trimester		2.54 (1.67, 3.12)		2.76 (2.01, 3.47)^***^
Site of antenatal care	Governmental health facility		1		1
	Non-governmental Health facility		1.29 (0.78, 1.89)		1.02 (0.82, 2.02)
Told pregnancy	No		1		1
complication	Yes		3.41 (2.71, 4.02)		2.73 (2.10, 3.21)^**^
Health care provider	No		1		1
Support	Yes		2.22 (1.88, 3.11)		1.77 (1.02, 2.44)^***^
Residence	Rural			1	1
	Urban			0.45 (0.34, 1.00)	0.71 (0.66, 1.06)
Region	South			1	1
	Central			1.01 (0.87, 1.17)	1.02 (0.87, 1.19)
	East			2.12 (1.56, 2.86)	1.55 (1.11, 2.44)^**^
	West			2.13 (1.56, 3.29)	2.01 (1.88, 2.76)^***^
Random effect	Community level variance (SE)	0.81 (0.01)	0.46 (0.02)	0.43 (0.01)	0.35 (0.02)
	ICC (%)	**18.2%**	6%	5.2%	3.5%
	MOR	**2.33 (2.28, 2.52)**	1.75 (1.42, 1.82)	1.69 (1.54,1.91)	1.52 (1.42, 1.61)
	PCV	Reference	43%	46%	**57%**
Model fit statistics	Log-likelihood	−81,558	−68,702	−70,926	−62,702
	Deviance	163,116	137,404	141,852	**125, 404**

*Significant at

** *p < 0.01 and*

*** *p < 0.001.*

*AOR, adjusted odds ratio.*

*CI, confidence interval.*

*Model 0, Empty (null) model.*

*Model I, Only individual-level explanatory variables included in the model.*

*Model II, Only community-level explanatory variables included in the model.*

*Model III, Combined model; both individual-level and community-level. explanatory variables.*

*PCV, proportional change in variance.*

*MOR, median odds ratio.*

## Discussion

The maternity continuum of care is the model of connecting pregnant women to ANC services, health facility delivery with skilled providers, and postpartum care within the recommended time limit (8). Low- and middle-income regions need to give attention to this priority area to achieve the sustainable development goal entitled, ending preventable maternal, neonatal, and child death by 2030 (21). Despite its contribution to the reduction of maternal and neonatal death evidence on the coverage of completion of the maternity continuum of care at the regional level (sub-Saharan Africa) is limited. Therefore, the current study was aimed to assess the pooled prevalence of completion of maternity continuum of care and its determinants in 33 sub-Saharan African countries.

In this study, the pooled prevalence of completion of the maternity continuum of care among 33 sub-Saharan African countries is found to be 35.81%; 95% [35.64%, 35.9%].

The findings of the current study are lower than the evidence from the previous studies in Motta and Hulet Eji Enese, Ethiopia 47% (8), Debre Berhan, Ethiopia 37.2% (22), Enemy District, Northwest Ethiopia 45% (15), and in India 39% (23). An explanation for this lower prevalence in the current study might be due to the difference in the composition of the study participants. The current study is based on the pooled prevalence from the DHS report of multiple countries, which has a higher proportion of rural communities and uneducated study participants, whereas the previous studies were based on a single city-based study setting with a higher proportion of educated study participants. This might inflate the prevalence compared with the current study, as education is one factor significantly associated with the outcome variables. On the other hand, the current study finds a higher prevalence compared with the previous study done at Arbaminch Ethiopia 9.5% (24), in Ethiopia from EDHS data analysis 13% (16), in rural women Ethiopia 13.9% (25), Nigeria 18% (26), Ghana 8% (27), and Tanzania 10% (28). The possible explanation for the higher prevalence of completion of the maternity continuum of care compared with the previous study done in Ethiopia might be due to the variation in the demarcation of the operational definition of the outcome variable. The current study has used all women who had at least one AMC visit, health facility delivery, and post-natal care in the recommended time, whereas the previous studies had used all women who had four and above ANC visits, skilled delivery, and postpartum care in the recommended time. This might inflate the prevalence in the current study as women with at least one ANC visit are included in the study. Finally, the possible explanation for the higher prevalence of completion of the maternity continuum of care in the current study compared with evidence from Ghana and Tanzania might be due to the high practice of traditional birth attendants in Ghana. There is evidence that shows people in Ghana are highly preoccupied with traditional beliefs, as health is determined by supper natural physical state, and less attention is given to healthcare provider induced recommendations including maternal healthcare services (29, 30).

Regarding the predictors of completion of the maternity continuum of care in the current study, ten variables (educational status of the women, wanted pregnancy, history of a terminated pregnancy, distance to the nearby health facility, decision-maker to attend maternity continuum of care, medication use during pregnancy, ANC starting, told pregnancy complication, health care provider support during ANC, and region) were statistically significant.

Participants with higher educational status are 3.61 times more likely to complete the maternity continuum of care compared with those who have no formal education. This finding is supported by different studies (8, 22, 23). The possible explanation might be due to an increased level of understanding of medical terms and cooperation with healthcare providers among educated women. Another possible explanation might be due to information about the importance of maternal healthcare services by healthcare professionals among educated women might make them less involved on traditional birth attendants compared with uneducated women influenced by the culture of the society.

Women with wanted pregnancies are 2.51 times more likely to complete the maternity continuum of care compared with women with an unwanted pregnancy. The findings of this study are similar to the previous study (15). This might be due to the higher health-seeking behavior of women with wanted pregnancies (31).

The odds of completing the maternity continuum of care is 3.21 times among women with a history of terminated pregnancy compared with those who have no history of terminated pregnancy. This might be because of the previous negative experience of pregnancy making them cautious about the current pregnancy outcome (32), and they might be fully involved across the continuum of care. The other possible explanation might be the effect of healthcare providers' intervention among those with high-risk pregnancies might increase the health-seeking behavior (33) and this might achieve completion of the maternity continuum of care.

Women with distance to the nearby health facility was not a big problem to attend the maternity continuum of care 2.11 times more likely to complete the maternity continuum of care compared with those whose distance to the nearby health facility was a big problem. The finding is in line with the previous studies (8). Distance is one factor that is under the group of delay two from the three-delay model to keep a woman safe throughout pregnancy, delivery, and postpartum period (34). Therefore, the longer the distance to the nearby health facility, the higher the dropout from the maternity continuum of care.

Independent decision-making women to attend the maternity continuum of care contributes 2.15 times higher to the completion of the maternity continuum of care as compared with women whose decisions were made by others. This finding is consistent with different studies (8, 15). This might be due to women who can decide independently to be able to easily avail themselves based on the schedule of the healthcare provider. The other hidden challenge of women who cannot decide by themselves mostly associated with an unwanted pregnancy and they might not give attention to the health status of their conception.

Women who did not use traditional medication during pregnancy were 1.01 times more likely to complete the maternity continuum of care compared with women who used traditional medication during pregnancy. The possible explanation might be due to women with experienced traditional medication highly adhered to the culture of the society, and their delivery process also attended by traditional birth attendants (35).

Women who had started ANC within the second trimester were 2.76 times more likely to complete the maternity continuum of care compared with those who started above the second trimester of gestational age. This finding is supported by the evidence (8, 22). ANC is the noteworthy predictor of the subsequent elements of the maternity continuum of care (36). Therefore, starting ANC in the early gestational age can make them informed about the importance of maternal healthcare services during pregnancy, delivery, and the postpartum period (8, 37), and women might attend the full components of the maternity continuum of care.

Participants who had been told about pregnancy complications are 2.73 times more likely to complete the maternity continuum of care as compared with those who had not been told about pregnancy complications. The finding of this study is conclusive to the previous study stated as the odds of completion of the maternity continuum of care were higher among women informed about danger signs/pregnancy complications (22, 25). The possible explanation could be due to the health-seeking behavior of women with information about pregnancy complications might be higher compared with those with no information (38) and this makes them conscious to fully engage across the continuum of care.

The odds of completing the maternity continuum of care among women with healthcare provider support during ANC are 1.77 times higher compared with those who did not have healthcare provider support. The possible explanation might be due to a positive attitude toward healthcare provider's appointments among women with healthcare provider support (39), and they might have health-seeking behavior due to clear communication with their healthcare provider.

Women in the East and West Africa are 1.55 and 2.01 times more likely to complete the maternity continuum of care as compared with women in the southern Africa, respectively. This might be due to the variation inaccessibility of maternal healthcare services. There is evidence that shows women in the southern Africa region are highly practicing traditional birth attendants (40), which may imply a lack of access to complete the maternity continuum of care.

## Strength and Limitations of the Study

The main strength of this study was the use of weighted nationally representative data with a large sample, which makes it representativeness at national and regional levels. Therefore, it can be generalized to all postpartum period women during the study period in Ethiopia. Moreover, the use of a multilevel model that took into account the hierarchical nature of the EDHS data and the variability within the community to get a reliable estimate and standard errors (SEs). However, it is not free of limitations mainly resulted from the use of secondary data. As some important confounders, such as the health service quality and behavioral factors are missed.

The other limitation of this study is considering women with at least one ANC visit for the completion of the maternity continuum of care.

## Conclusions

The pooled prevalence of completion of the maternity continuum of care was found to be low. Educational attainment, college and above, wanted pregnancy, history of a terminated pregnancy, the distance was not a big problem, women primary decision-maker, not exposed to traditional medication, ANC starts within the second trimester, told about pregnancy complication, healthcare provider support, eastern Africa, and western Africa were positively associated with the completion of the maternity continuum of care.

## Data Availability Statement

The original contributions presented in the study are included in the article/supplementary material, further inquiries can be directed to the corresponding author.

## Ethics Statement

Ethical review and approval was not required for the study on human participants in accordance with the local legislation and institutional requirements. Written informed consent for participation was not required for this study in accordance with the national legislation and the institutional requirements.

## Author Contributions

MH and DB have conceptualized and developed the proposal, analyzed the data, written the manuscript, revised and edited the final version, and approved it for submission to the journal. Both authors contributed to the article and approved the submitted version.

## Conflict of Interest

The authors declare that the research was conducted in the absence of any commercial or financial relationships that could be construed as a potential conflict of interest.

## Publisher's Note

All claims expressed in this article are solely those of the authors and do not necessarily represent those of their affiliated organizations, or those of the publisher, the editors and the reviewers. Any product that may be evaluated in this article, or claim that may be made by its manufacturer, is not guaranteed or endorsed by the publisher.

## Abbreviations

ANC: antenatal care
AOR: adjusted odds ratio:
CI: confidence interval
DHS: Demographic and Health Survey
ICC: intraclass correlation coefficient
MOR: median odds ratio
PCV: proportion change in variance.
